# The influence of microsatellite polymorphisms in sex steroid receptor genes ESR1, ESR2 and AR on sex differences in brain structure

**DOI:** 10.1016/j.neuroimage.2020.117087

**Published:** 2020-11-01

**Authors:** Geoffrey Chern-Yee Tan, Carlton Chu, Yu Teng Lee, Clarence Chih King Tan, John Ashburner, Nicholas W. Wood, Richard SJ. Frackowiak

**Affiliations:** a Institute of Mental Health, National Healthcare Group, Singapore; b Clinical Imaging Research Centre, National University of Singapore, Singapore; c DeepMind Technologies Limited, United Kingdom, UK; d Wellcome Centre for Human Neuroimaging, University College London (UCL), United Kingdom, UK; e Biomedical Research Centre, UCL, UK; f Laboratoire de Recherche en Neuroimagerie, CHUV University Hospital, Switzerland; g Singapore Institute for Clinical Sciences, Agency for Science, Technology and Research (A^∗^STAR), Singapore; i University of Sydney, Sydney, NSW, Australia

## Abstract

The androgen receptor (AR), oestrogen receptor alpha (ESR1) and oestrogen receptor beta (ESR2) play essential roles in mediating the effect of sex hormones on sex differences in the brain. Using Voxel-based morphometry (VBM) and gene sizing in two independent samples (discovery n ​= ​173, replication ​= ​61), we determine the common and unique influences on brain sex differences in grey (GM) and white matter (WM) volume between repeat lengths (n) of microsatellite polymorphisms AR(CAG)n, ESR1(TA)n and ESR2(CA)n. In the hypothalamus, temporal lobes, anterior cingulate cortex, posterior insula and prefrontal cortex, we find increased GM volume with increasing AR(CAG)n across sexes, decreasing ESR1(TA)n across sexes and decreasing ESR2(CA)n in females. Uniquely, AR(CAG)n was positively associated with dorsolateral prefrontal and orbitofrontal GM volume and the anterior corona radiata, left superior fronto-occipital fasciculus, thalamus and internal capsule WM volume. ESR1(TA)n was negatively associated with the left superior corona radiata, left cingulum and left inferior longitudinal fasciculus WM volume uniquely. ESR2(CA)n was negatively associated with right fusiform and posterior cingulate cortex uniquely. We thus describe the neuroanatomical correlates of three microsatellite polymorphisms of steroid hormone receptors and their relationship to sex differences.

## Highlights

• Microsatellite polymorphisms in sex hormone receptor genes influence volume in regions of brain sex difference
• AR(CAG)n repeat length is positively associated with grey and white matter volume across males and females
• ESR1(TA)n repeat length is negatively associated with grey and white matter volume across males and females
• ESR2(CA)n repeat length is negatively associated with grey matter volume in females but not in males
• Repeat length was associated with volume in the hypothalamus, insula, temporal cortices, prefrontal cortices, inferior and superior longitudinal fasciculi in the three genes.
• These regions were largely replicated in an independent cohort acquired on a separate scanner.

## 1 Introduction

### 1.1 Sex differences

Sex differences in brain structure are well-established across a range of studies. Males are also known to have a larger brain; particularly in the amygdala, hypothalamus, cerebellum and temporal lobe (Cosgrove et al., 2007; Good et al., 2001; Ruigrok et al., 2014; Witte et al., 2010). Females appear to have proportionally more grey matter (GM) overall and across the cortex, such as in the parietal cortex, planum temporale, Heschl’s gyrus and anterior cingulate gyrus (Cosgrove et al., 2007; Gur et al., 1999; Luders et al., 2006; Ruigrok et al., 2014). Recently, a UK Biobank study of 5216 participants found multiple brain sex differences – including higher uncorrected volumes and surface areas in males and higher cortical thickness and white matter (WM) tract complexity in females. Specific regions, such as the right insula, were larger in males after correction, while areas like the superior parietal were larger in females (Ritchie et al., 2018). It has been argued that some of these differences are accounted for by differences in brain size and the allometric relationship between brain size and GM proportion (Zhang et al., 2000). However burgeoning evidence suggests that many of these differences are attributable to specific biological factors.

### 1.2 Sex hormones

In vivo characterisation of the influences of the biological determinants of brain sex differences can be a challenge due to the multiple confounds of sex. Two major influences that may account for brain sex differences are differing expression of genes from the sex chromosomes and the action of sex steroid hormones, such as androgens and oestrogen (Hines, 2010, 2011).

Androgens have been identified as important for brain development. Foetal testosterone levels positively predict right temporoparietal junction GM and negatively predict planum temporale volume congruent with their brain sex differences (Lombardo et al., 2012). Administration of testosterone to female-to-male transgender individuals was shown to be associated with reductions in grey matter volume in Broca’s and Wernicke’s areas as well as the mean diffusivity of their white matter connection in the external capsule (Hahn et al., 2016). Endogenous testosterone affects brain development across childhood and adolescence in regions such as the amygdala, hippocampus and parietal cortex differently between the sexes (Nguyen et al., 2013; Neufang et al., 2009; Nguyen, 2018; Wierenga et al., 2018).

Oestrogen has been associated with sex differences in the development of brain regions such as the cerebellum (Hedges et al., 2012) and hypothalamus (Lenz and McCarthy, 2010). In pubertal girls, serum estradiol is associated with increased GM in frontal, interior temporal and occipital gyri and decreased GM in prefrontal, parietal and temporal regions (Peper et al., 2009). Administration of exogenous oestrogen has been found to increase brain ventricular volume in menopausal women (Kantarci et al., 2016) as well as in male-to-female transgender individuals, associated with a corresponding decrease in regional brain matter (Seiger et al., 2016; Zubiaurre-Elorza et al., 2014).

Sex hormones act through nuclear and membrane receptors in the brain to induce wide-ranging changes in transcription, development, plasticity and neural signaling. However, the overlap in activity of sex hormones and sex hormone receptors makes inferring the respective influence of each hormone difficult. Any given sex hormone acts on several receptors, both in the nucleus and outside of it. Furthermore, a degree of cross-receptor activity exists between receptors. For example, androgen receptors inhibit estrogen receptor activity when coexpressed on the same cell, and low levels of androgen receptors are prognostic of breast cancer (Peters et al., 2009). Furthermore, androgens are able to bind and cross-activate the oestrogen receptor (Garcia and Rochefort, 1979) and are converted to oestrogen by aromatization (Lephart, 1996). Thus it is of value to distinguish between effects related to sex hormone receptors and those related to sex hormone levels although they lie on a common pathway.

### 1.3 Sex hormone receptor genetics

An alternative approach to investigating the effect of sex hormones in the brain makes use of individual variation in genotypes influencing the expression of the androgen receptor and oestrogen receptor. This approach of utilising common genetic variation to determine molecular influences in the brain has been validated in a number of previous studies involving this cohort and subgroups selected from it (Wittman et al., 2013; Tan et al., 2010; Sebastian et al., 2010; Roiser et al., 2008).

#### 1.3.1 Androgen receptor (AR) gene

The primary effects of androgens occur through the activation of the androgen receptor, a nuclear transcription factor encoded by the AR gene on the X-chromosome (Choong et al., 1996). The AR gene contains a CAG repeat polymorphism within its first exon coding for a polyglutamine tract of variable length (Chamberlain et al., 1994). Longer repeats of this polymorphic polyglutamine tract in the N-terminal exon of AR inhibit its interaction with co-activators and the transcription of its gene to mRNA (Beilin et al., 2000; Chamberlain et al., 1994; Choong et al., 1996). Men with a mutation in AR(CAG)n (where n refers to the number of CAG repeats, n ​> ​38) manifest Kennedy’s disease or spinobulbar muscular atrophy (La Spada et al., 1991, 1992), while low n alleles have been associated with risk for prostate cancer and benign prostatic hyperplasia (Giovannucci et al., 1999; Kumar et al., 2011; Qin et al., 2016). In women, AR(CAG)n has been associated with testosterone levels (Westberg et al., 2001), bone mineral density (Yamada et al., 2005) and obesity (Gustafson et al., 2003) as well as cancers of the breast and ovaries (Deng et al., 2017; Hao et al., 2010).

AR(CAG)n has also been associated with brain development, with lower n found to interact with testosterone level to predict greater increase in relative white-matter volume (Perrin et al., 2008) and greater decrease in relative grey-matter volume (Paus et al., 2010) in male adolescents. In female adolescents, AR(CAG)n has been positively associated with rate of cortical thinning in the inferior frontal gyrus instead (Raznahan et al., 2010). Furthermore, AR(CAG)n has been negatively associated with intellectual giftedness (Celec et al., 2013) as well as general cognitive functioning as assessed by performance on tests of general cognition and processing speed (Yaffe et al., 2003) in males; and has been found to interact with testosterone to predict performance on the Morris water maze differentially across the sexes as well (Nowak, Diamond, Land & Moffat, 2014). It has also been associated with aspects of personality, including extraversion (Westberg et al., 2009) and aggression (Butovskaya et al., 2015). AR(CAG) also associates with cognitive decline and increased brain atrophy in elderly adults, demonstrating the influence of AR(CAG) on the brain (Gardiner et al., 2019).

#### 1.3.2 Oestrogen receptor α (ESR1) gene

The two main nuclear receptors of oestrogen are oestrogen receptor α and β - encoded by the ESR1 and ESR2 genes respectively. The α subtype is well-known to influence the brain and is expressed in most regions, with particularly high expression within the amygdala and hypothalamus (Laflamme et al., 1998). The TA microsatellite repeat polymorphism, located in the promoter region upstream of ESR1, is in linkage disequilibrium with a number of other putative transcription binding sites and influences transcription functionally (Becherini et al., 2000; Langdahl et al., 2000; Prichard et al., 2002). Evidence for the functionality of the TA polymorphism is further supported through its association with a number of oestrogen-related clinical phenotypes such as female adult stature (Schuit et al., 2004), bone mineral density (Langdahl et al., 2000) and endometriosis (Zhao et al., 2016).

ESR1(TA)n (where n refers to repeat number) has been associated with higher and more feminine left hand 2D:4D digit ratios in men (Vaillancourt et al., 2012). The 2D:4D ratio is frequently used as a proxy of prenatal hormone levels (Valla and Ceci, 2011) and has been positively associated with volume of cerebral cortex, total cerebellar cortex and total cerebellar WM in males (Darnai et al., 2016) as well as negatively associated with volume of dorsal anterior cingulate cortex GM in females (Gorka et al., 2015).

#### 1.3.3 Oestrogen receptor β (ESR2) gene

ESR2, like ESR1, contains multiple polymorphisms that have been implicated in diseases related to old age and reproduction, as related to bone mineral density (Ichikawa et al., 2005), cancers of the breast and ovary (Tang et al., 2018; Yu et al., 2011) and hypertension and cardiovascular risk (Ogawa et al., 2000; Rexrode et al., 2007). Additionally, however, it has been associated with Alzheimer’s disease (Pirskanen et al., 2005), Parkinson’s disease (Westberg et al., 2004), chronic fatigue syndrome (Gräns et al., 2007), anorexia nervosa (Eastwood et al., 2002) and bulimia (Nilsson et al., 2004).

In particular, ESR2 has a 5’ flanking region containing a number of regulatory elements, including a CA repeat microsatellite polymorphism that influences expression of the β receptor (Tsukamoto et al., 1998). This region is also relatively GC-rich and could be expected to be susceptible to methylation. Shorter ESR2(CA)n (where n refers to the number of CA repeats) has been found to associate with increased androgen levels (Westberg et al., 2001), with there being some support of a similar dosage effect with oestrogen levels as well (Scariano et al., 2004).

### 1.4 Aims

Whilst these polymorphisms in genes of the sex hormone pathways have been associated with individual differences in cognition and personality, it is still unclear how they influence cortical brain structure and whether they explain related sex-associated inter-individual differences.

Whereas previous structural imaging studies suggest an influence of polymorphisms of AR(CAG)n on the changes in GM and WM volume in adolescence (Paus et al., 2010; Perrin et al., 2008; Raznahan et al., 2010), this study seeks to clarify the effect on the brain of these three polymorphisms across different brain regions. In the present study, we investigated the influence of polymorphic AR(CAG)n, ESR1(TA)n and ESR2(CA)n on human brain structure by examining T1-weighted images from a large sample of healthy volunteers using computational neuroanatomical techniques. Our hypothesis is that the brain regions covarying in volume with sex hormone polymorphisms would be a subset of brain regions that differ between the sexes.

## 2 Methodology

### 2.1 Recruitment

Healthy previous volunteers at the Wellcome Trust Centre for Neuroimaging at University College London were screened for any previous neurological or psychiatric conditions via administration of the Mini-International Neuropsychiatric Interview (MINI). The interview was administered by one of the paper authors, who has been trained in the administration of the interview. All available scans of subjects meeting these criteria were used. Subjects gave written informed consent and the study was approved by Great Ormond Street Hospital for Children NHS Trust and Institute of Child Health, Research Ethics Committee (07/Q0508/32**)**.

### 2.2 Genotyping

#### 2.2.1 AR(CAG)n

For each individual, DNA was extracted from peripheral lymphocytes using standard techniques. A 370–450 bp fragment was amplified by PCR with a FAM-labeled forward primer.

AR1 FAM-5′-GCCTGTTGAACTCTTCTGAGC-3′,

AR2 5′ GCTGTGAAGGTTGCTGTTCCTC-3’

Amplification was performed in 33 cycles with a denaturation temperature of 95 ​°C for 30s, an annealing temperature of 55 ​°C for 30s and an extension temperature of 72 ​°C for 30s, with a final extension of 72 ​°C for 10min. 1 ​μL of PCR product was added to 9 ​μL of formamide with 0.3 ​μL of LIZ-500 standard, denatured at 95 ​°C for 5min and placed on ice.

#### 2.2.2 ESR1(TA)n

A 160–194 bp fragment was generated with a FAM-labeled forward primer.

ESR1F FAM-5′-GACGCATGATATACTTCACC-3′,

ESR1R 5′-GCAGAATCAAATATCCAGATG-3’.

Amplification was performed in 28 cycles with a denaturation temperature of 95 ​°C for 30s, an annealing temperature of 58 ​°C for 30s and an extension temperature of 72 ​°C for 30s, with a final extension at 72 ​°C for 10min. 1 ​μL of PCR product was added to 9 ​μL of formamide with 0.3 ​μL of LIZ-500 standard, denatured at 95 ​°C for 5min and placed on ice.

#### 2.2.3 ESR2(CA)n

A 147–187 bp fragment was generated with a HEX-labeled forward primer.

ESR2F HEX-5′- GGTAAACCATGGTCTGTACC -3’.

ESR2R 5′- AACAAAATGTTGAATGAGTGGG -3’.

Amplification was performed in 35 cycles with a denaturation temperature of 95 ​°C for 30s, an annealing temperature of 62 ​°C for 45s and extension temperature of 72 ​°C for 60s, with a final extension at 72 ​°C for 10min. 1 ​μL of PCR product was added to 9 ​μL of formamide with 0.3 ​μL of LIZ-500 standard, denatured at 95 ​°C for 5min and placed on ice. All DNA was analysed on the ABI 3730 DNA sequencer equipped with Genescan (ABI, Warrington, UK) software.

### 2.3 Imaging

#### 2.3.1 Discovery cohort image acquisition

Scanning was performed on a Sonata 1.5T whole body scanner (Siemens Medical Systems) using a whole body coil for transmission and 8-channel phased array head coil for reception, using a 3D-Modified Driven Equilibrium Fourier Transform (3D-MDEFT) sequence (Deichmann et al., 2004), with FLASH-EPI hybrid readout (Deichmann et al., 2006) TR 20.66 ​ms, TE 8.46 ​ms, FA 25°, 1 ​mm isotropic, image dimensions 240 ​× ​256 ​× ​176, total duration 8 ​min.

#### 2.3.2 Replication cohort image acquisition

Scanning was performed on an Allegra 3.0T scanner, using a 3D-Modified Driven Equilibrium Fourier Transform (3D-MDEFT) sequence with a standard transmit-receive coil (Deichmann et al., 2004), TR 7.92 ​ms, TE 2.4 ​ms, FA 15°, 1 ​mm isotropic, image dimensions 240 ​× ​256 ​× ​176, total duration 8 ​min.

#### 2.3.3 Image preprocessing

Images were screened by both radiographers and researchers for image quality and artifacts and were screened for abnormalities by neuroradiologists. Images were analysed using Voxel-Based Morphometry (VBM) in SPM12 (Wellcome Trust Centre for Neuroimaging). Segmentation into GM, WM and cerebrospinal fluid was performed using unified segmentation (Ashburner and Friston, 2005). A high dimensional warp using a fast-diffeomorphic algorithm was used (Ashburner, 2007) to spatially normalise the tissue maps with modulation.

### 2.4 Analysis

#### 2.4.1 Cohort information

The discovery sample comprises of 200 participants, 107 females and 93 males. The participants ranged from 16 to 75 years old with mean age ​= ​32.4 years, SD ​= ​12.6. Mean grey volume for the discovery sample is 708.6, SD ​= ​169.8. The replication sample comprises of 72 participants, 39 females and 33 males. The participants ranged from 18 to 74 years old with mean age ​= ​29.9, SD ​= ​11.0. Mean grey volume for the replication sample is 705.5, SD ​= ​123.6. Not every participant was retained for analysis – we filtered out non-Caucasians from the both samples for VBM as a confounder as it has been demonstrated that genetic polymorphisms exert different influences on phenotype in different races. The sample descriptives are presented in Table S1 in the Supplementary Information after non-Caucasian individuals were filtered from the sample.

To test for any significant differences between the groups, a one-way ANOVA was conducted with age, grey volume, white volume, AR(CAG)n, ESR1(TA)n, ESR2(TA)n across the filtered discovery and replication samples. The means were not significantly different between the groups across all of the measures, suggesting that the two groups are not significantly different across those measures.

Demographic information such as age and sex, known confounds of brain volume, and medical history, were collected. Information on prescription medications were also collected from participants. 11 women were on oral contraceptives or hormone replacement therapy. Participant medical history was screened for medical conditions that would affect cell response to androgens, such as Complete Androgen Insensitivity Syndrome, and there were no participants excluded from medical histories. 1 participant was excluded because chromosomal sex did not match reported gender. 13 individuals were left-handed.

As genotypes were microsatellites, it was not possible to make group comparisons however genotype histograms of repeat length are attached as a supplement for reference. Results of one-way ANOVA on participant characteristics are presented in Table S2 in the Supplementary Information.

#### 2.4.2 Discovery sample

To identify regions of the brain that were different between males and females, sample images were contrasted via sex. Modulated, normalised GM maps, derived from structural magnetic resonance scans, were smoothed and compared by VBM. The discovery sample comprises of 200 participants. Of the 200 participants, 27 participants were non-Caucasian and were excluded from the analysis. 3 participants had errors in ESR1(TA)n genotyping and were also excluded from VBM. Thus, the final sample sizes for VBM for AR(CAG)n, ESR1(TA)n, and ESR2(CA)n are 173, 170, 173 respectively.

Regions from the comparison were subsequently compared with regions shown in subsequent analysis by genotype. VBM analysis was performed with each polymorphism independently for AR(CAG)n, ESR1(TA)n and ESR2(CA)n. A median split was used for microsatellite polymorphisms to divide alleles into high and low repeats. Thus, genotype was defined as a three-level condition, grouping by short homozygotes, heterozygotes and long homozygotes. As the AR(CAG)n polymorphism is on the X-chromosome, there were no male heterozygotes. An additive effect of repeat length was found and a continuous variable was calculated by averaging the two allele repeat lengths. Sex was used as an independent condition, while age, GM and WM volume were used as covariates after Gram-Schmidt orthogonalisation. Individual t-tests were conducted to determine the effect of sex and genotype applying family-wise error thresholds for peak and cluster-level results. Due to the large number of statistical tests conducted across 3 gene polymorphisms, a Bonferroni correction for multiple testing was performed at p ​= ​0.05/3 ​= ​0.0166. All results significant post-multiple correction are highlighted in their respective tables.

In order to determine regions overlapping with regions of sex difference, a mask was created from the thresholded statistical parametric map (p ​< ​0.05) for regions larger in males and for regions larger in females. Analyses were repeated using a threshold of p ​< ​0.001 using these masks and coordinates are reported to have a conjunction where they were found to lie in regions of brain sex differences.

All regions reported in the results were statistically significant at p ​< ​0.05 after a family-wise error correction across the whole-brain level. Significance at the voxel-level was thresholded at p ​< ​0.001.

#### 2.4.3 Replication sample

The replication sample comprised 72 subjects whose images were acquired independently on a different scanner. This sample was analysed independently as it has been shown that scanner effects has both main and interaction effects on associations in VBM (Stonnington et al., 2008). Of the sample, 11 participants were non-Caucasian and were filtered out, and 1 participant had errors in ESR1(TA)n genotyping and was excluded from VBM. The sample sizes for AR(CAG)n, ESR1(TA)n, and ESR2(CA)n for VBM are 61, 60, 61 respectively.

In the replication cohort, the thresholded mask (p ​< ​0.05 uncorrected) of the voxels significantly associated with GM volume with the AR(CAG)n, ESR1(TA)n and ESR2(CA)n polymorphisms in the discovery cohort was used to determine whether these regions were also significantly associated with the respective polymorphisms in an independent cohort scanned on another scanner. After preprocessing, identical contrasts were used to determine whether the same regions were associated with genetic variation. All regions reported in the results were statistically significant at p ​< ​0.05 after family-wise error correction on the whole brain level, unless stated otherwise. No Bonferroni correction was performed for replication as we tested *a priori* hypotheses. Due to limitations on publication length, the results of the replication cohort are presented in Supplementary Information.

## 3 Results

**Fig. 1:**
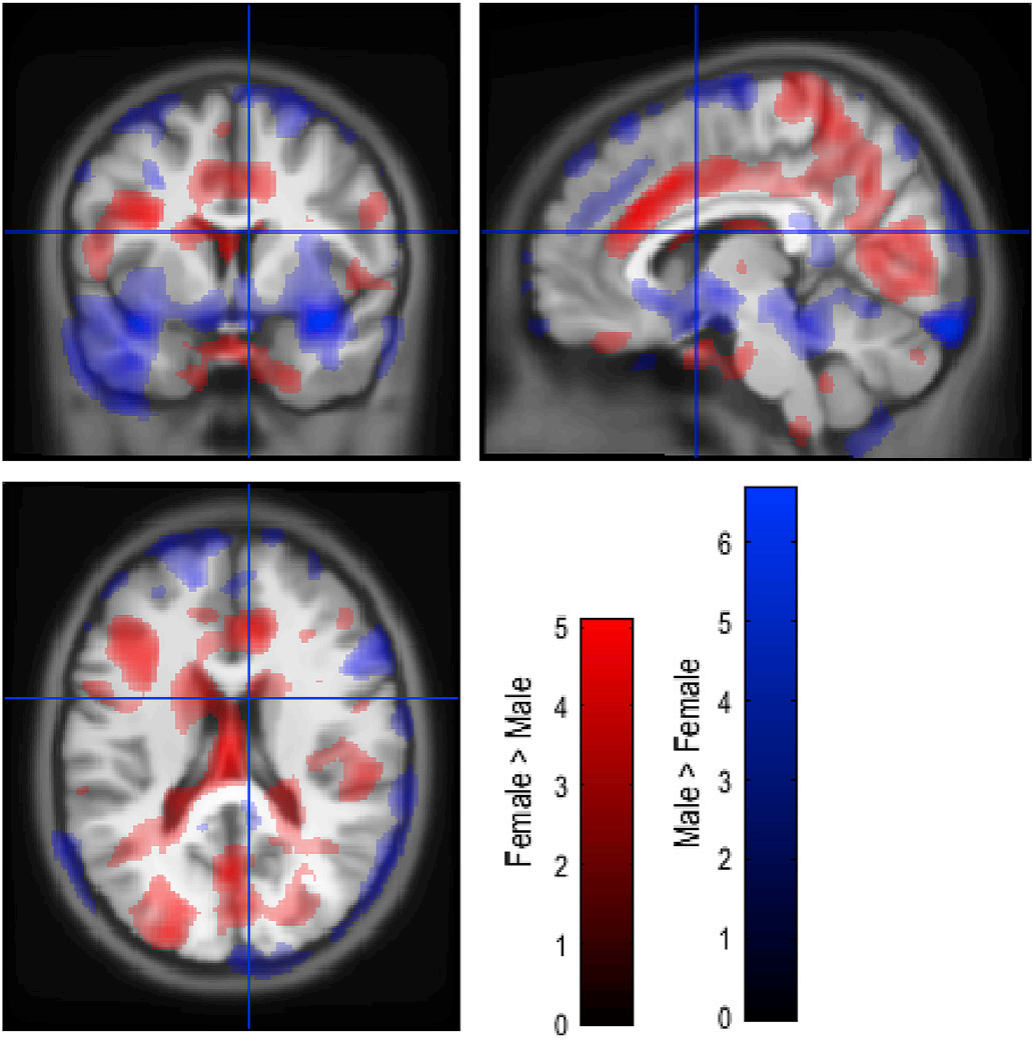
Regions showing sex differences in GM volume. Red shows regions with greater GM volume in females. Blue shows regions with greater GM volume in males. T-statistic maps thresholded at p ​< ​0.05 uncorrected were overlaid on the average of individual T1-weighted images warped together into MNI space.

**Fig. 2:**
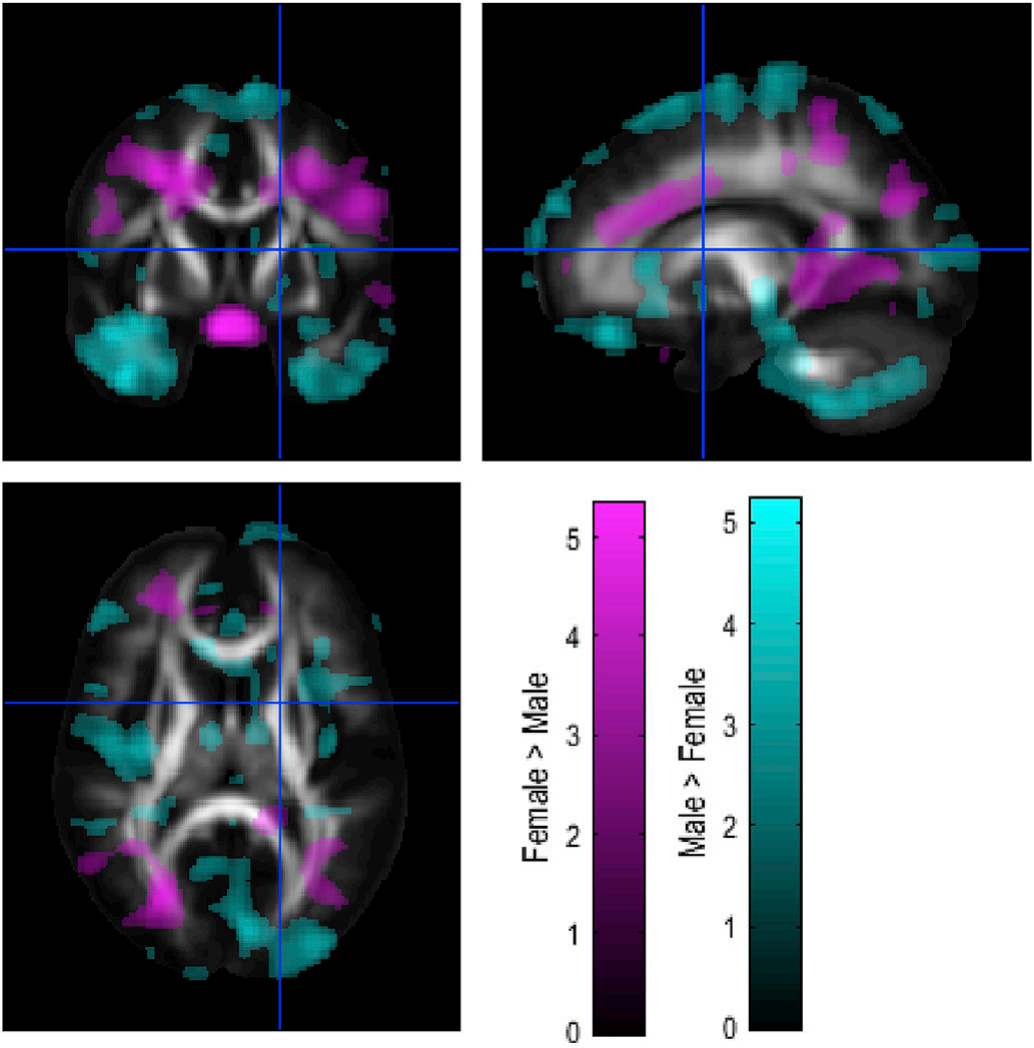
Regions showing sex differences in WM volume. Purple shows regions with greater WM volume in females. Turquoise shows regions with greater WM volume in males. T-statistic maps thresholded at p ​< ​0.05 uncorrected were overlaid on the average of individual T1-weighted images warped together into MNI space.

**Fig. 3:**
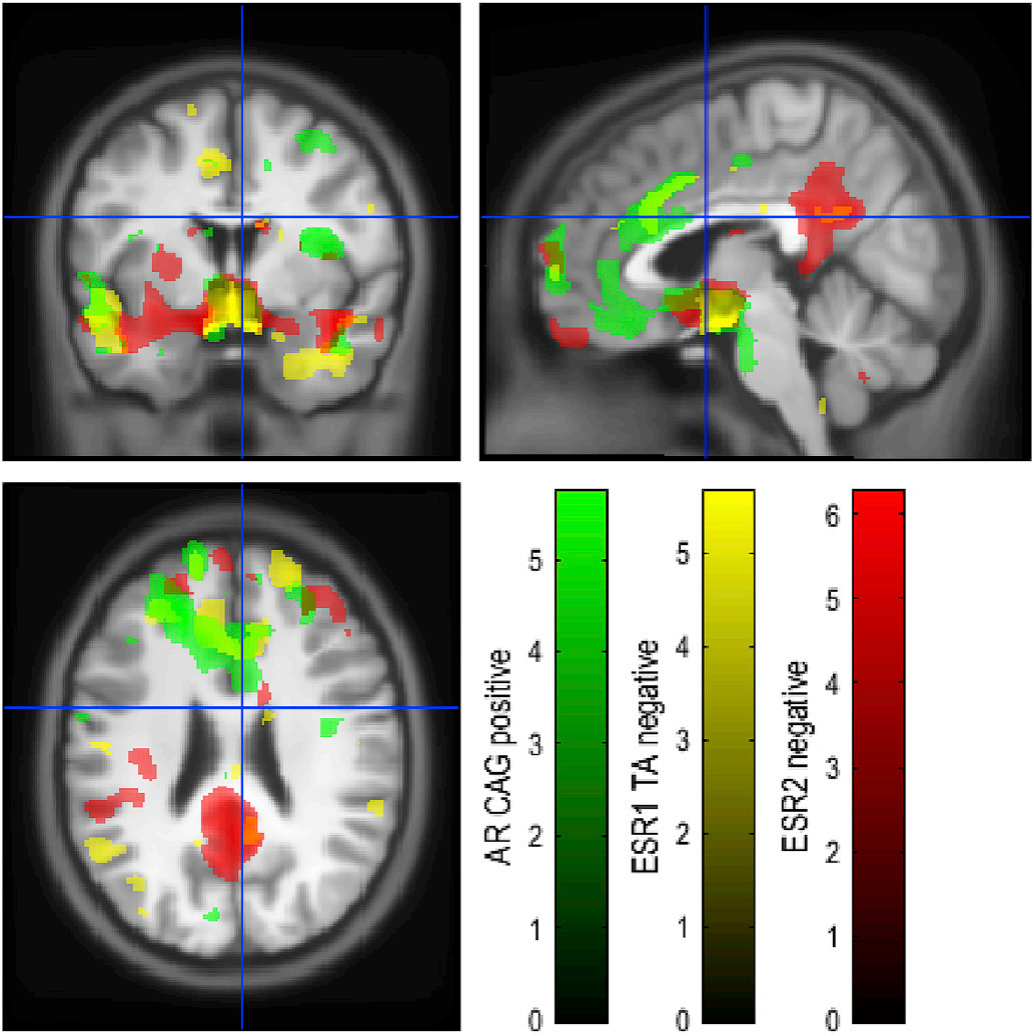
Regions associated with AR(CAG)n, ESR1(TA)n and ESR2(CA)n in GM volume. T-statistic maps thresholded at p ​< ​0.001 uncorrected were overlaid on the average of individual T1-weighted images warped together into MNI space.

**Fig. 4:**
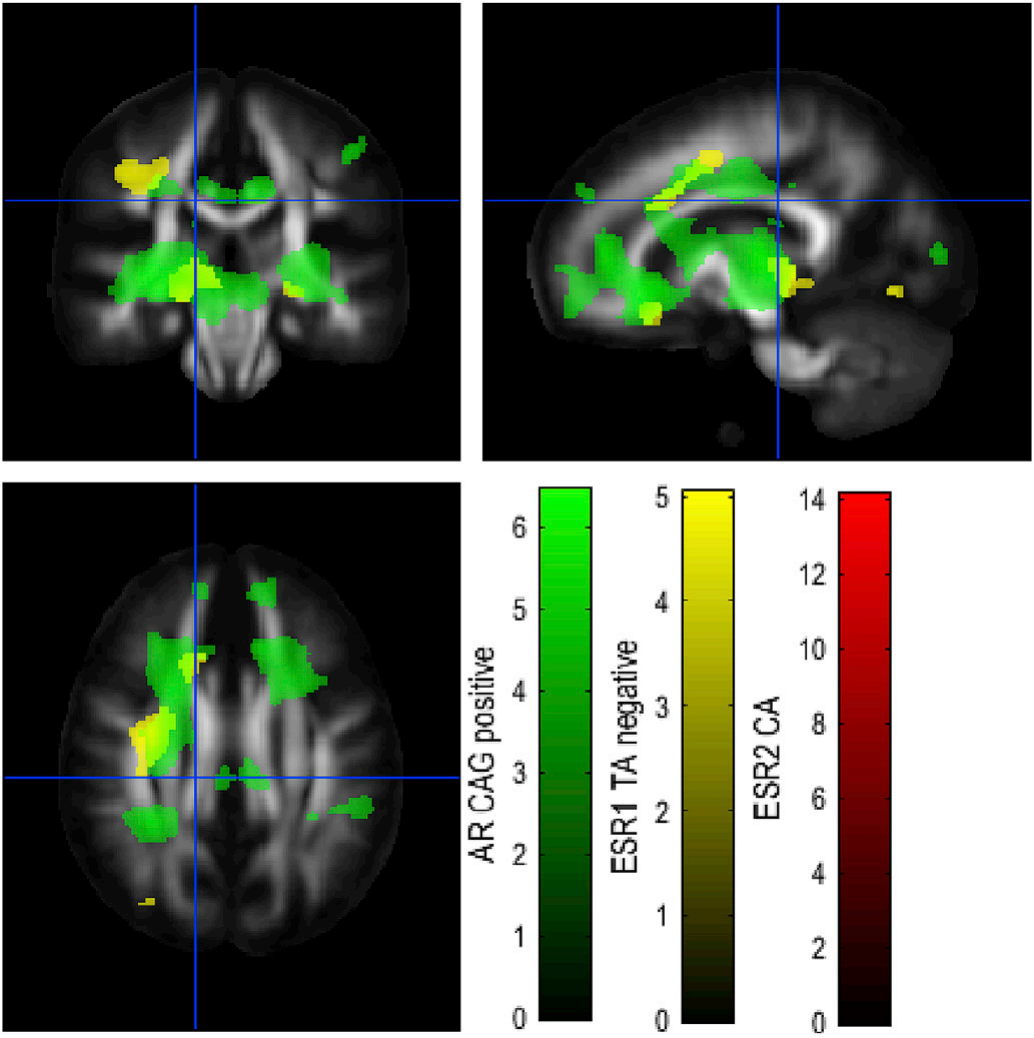
Regions associated with AR(CAG)n, ESR1(TA)n and ESR2(CA)n in WM volume. T-statistic maps thresholded at p ​< ​0.001 were overlaid on the ICBM FA template.

A regional map of sex differences in GM, Fig. 1 and Table S3 in Supplementary Information, and WM differences, Fig. 2 and Table S4 in Supplementary Information, was generated in order to determine overlap with genetic differences. Associations with genotype were then analysed, showing overlap between genotypic differences in GM (Fig. 3) and WM (Fig. 4). Associations with each microsatellite polymorphism were analysed in the discovery cohort for AR(CAG)n (Table 1, Table 2), ESR1(TA)n (Table 3, Table 4) and ESR2(CA)n (Table 5) as well as in the replication cohort (Tables S5-7 in Supplementary Information).

**Table 1:** GM regions showing statistically significant positive correlations with AR(CAG)n in the discovery sample, family-wise error corrected<0.05.

GM Regions with positive correlation with AR(CAG)n					
Region	[x, y, z] MNI	Z-score	p-value, cluster, FWE-corrected	p-value, peak, FWE-corrected	Conjunction with sex differences
L Anterior cingulate	−17, 39, 23	5.45	6.4 ​× ​10^−5^^a^	0.0032^a^	F ​> ​M
L Rostral prefrontal	−32, 54, 3	5.28	3.7 ​× ​10^−4^^a^	0.0077^a^	M ​> ​F
L Orbitofrontal	−14, 35, −15	5.04	0.0036^a^	0.023	M ​> ​F
R Cerebellum	18, −44, 44	5.00	0.017	0.029	M ​> ​F
L Dorsolateral prefrontal	−27, 27, 44	4.96	0.031	0.033	M ​> ​F
R Dorsolateral prefrontal	32, 39, 32	4.96	0.016^a^	0.033	M ​> ​F
L Ventrolateral prefrontal	−29, 41, −15	4.88	0.006^a^	0.049	M ​> ​F
L Middle temporal BA21	−56, −14, −20	4.86	0.040		M ​> ​F
L Medial prefrontal	−17, 48, 8	4.83	0.024		F ​> ​M
R Hypothalamus	9, −5, −14	4.82	4.4 ​× ​10^−4^^a^		M ​> ​F
L Hypothalamus	−8, −6, −11	4.82			M ​> ​F
R Posterior insula/planum temporale	42, −2, −26	4.79	0.0048^a^		M ​> ​F
R Rostral prefrontal	39, 57, 2	4.69	0.027		M ​> ​F

L: Left R: Right M: Male F: Female BA: Brodmann’s area.

a Significant after Bonferroni correction at p ​= ​0.05/3.

**Table 2:** WM regions showing statistically significant positive correlations with AR(CAG)n in the discovery sample, family-wise error corrected<0.05.

WM Regions with positive correlation with AR(CAG)n					
Region	[x, y, z] MNI	Z-score	p-value, cluster	p-value, peak	Conjunction with sex differences
L Anterior corona radiata	−24, 35, 8	6.08	3.6 ​× ​10^−10^^a^	1.1 ​× ​10^−4^^a^	F ​> ​M
L Superior Longitudinal Fasciculus	−23, 6, 21	5.83		4.4 ​× ​10^−4^^a^	F ​> ​M
	−21, 17, 18	5.42		0.0038^a^	F ​> ​M
L Thalamus	−21, −21, 5	5.60	1.0 ​× ​10^−7^^a^	0.0015^a^	
L Fornix/Stria terminalis, insula posterior long	−24, −26, −3	5.26		0.0082^a^	M ​> ​F
L Retrolenticular part of internal capsule, fusiform	−35, −26, 0	5.10		0.017	
R Anterior corona radiata	29, 36, 12	5.36	4.6 ​× ​10^−4^^a^	0.0051^a^	F ​> ​M
L Anterior corona radiata	−15, 24, −8	5.05	0.0040^a^	0.022	F ​> ​M

L: Left R: Right M: Male F: Female.

a Significant after Bonferroni correction.

**Table 3:** GM regions showing statistically significant negative correlations with ESR1 (TA) repeat length in the discovery sample, family-wise error corrected<0.05.

GM Regions with negative correlation with ESR1(TA)n					
Region	[x, y, z] MNI	Z-score	p-value cluster	p-value peak	Conjunction with sex differences
L Ventrolateral prefrontal	−38, 44, −9	5.38	2.1 ​× ​10^−4^^a^	0.0012^a^	F ​> ​M
	−38, 53, −11	5.08		0.0052^a^	
L Hypothalamus	−2, −3, −15	5.14	5.4 ​× ​10^−4^^a^	0.0040^a^	M ​> ​F
R Hypothalamus	8, −3, −20	5.02		0.0070^a^	M ​> ​F
L Middle and inferior temporal gyri	−62, 21, −8	5.08	0.0051^a^	0.0051^a^	M ​> ​F
L Posterior insula/planum temporale	−45, −5, −24	4.87		0.014^a^	M ​> ​F
L Cerebellum	−41, −65, −35	5.04	3.1 ​× ​10^−4^^a^	0.0062^a^	M ​> ​F
R Cerebellum	39, −68, −33	3.80	0.023		M ​> ​F
L Subgenual anterior cingulate	−12, 35, −14	4.89	0.037	0.012^a^	
R Anterior temporal	41, 3, −30	4.72		0.026	M ​> ​F
R Middle and inferior temporal gyri	63, −21, −17	4.71	0.0055^a^	0.027	M ​> ​F
L Anterior cingulate	−2, 24, 30	4.62	4.3 ​× ​10^−4^^a^	0.039	F ​> ​M
L Posterior temporal	−39, −62, −9	4.60	0.0065^a^	0.042	M ​> ​F
L Superior temporal	−41, −18, −8	4.59		0.044	M ​> ​F
L Superior frontal	−18, 33, 45	4.57	0.040	0.049	
L Rostral prefrontal	−6, 62, 11	4.33	0.026		

L: Left R: Right M: Male F: Female.

a Significant after Bonferroni correction.

**Table 4:** WM regions showing statistically significant negative correlations with ESR1 (TA) repeat length in the discovery sample, family-wise error corrected<0.05.

WM Regions with negative correlation with ESR1(TA)n					
Region	[x, y, z] MNI	Z-score	p-value cluster	p-value peak	Conjunction with sex differences
Left SLF	−33,-8,20	4.84	9.1 ​× ​10^−6^∗	0.0088∗	F ​> ​M
	−33,-17, 35	4.78		0.011∗	F ​> ​M
	−30, −12, 29	4.75		0.013∗	F ​> ​M
Left ILF, occipital	−15, −35, 0	4.65	0.019	0.020	F ​> ​M
L ILF, Temporal/Cingulum	32, −75, 6	4.54		0.031	

L: Left F: Female M: Male ILF: Inferior Longtitudinal Fasciculus SLF: Superior Longitudinal Fasciculus.

**Table 5:** GM regions showing statistically significant negative correlations with ESR2(CA)n in the discovery sample, family-wise error corrected<0.05.

GM Regions with negative correlation with ESR2(CA)n					
Region	[x y z] MNI	Z-score	p-value, cluster	p-value, peak	Conjunction with sex differences
R Posterior insula/planum temporale	44, 2, −18	5.92	7.5 ​× ​10^−6^^a^	2.8 ​× ​10^−4^^a^	M ​> ​F
	45, −9, −12	4.90		0.043	
L Rostral prefrontal	−32, 59, 0	5.65	5.7 ​× ​10^−5^^a^	0.001^2^^a^	M ​> ​F
	−33, 59, 14	4.99		0.030	
L & R Hypothalamus	−11, 0, −18	5.17	1.4 ​× ​10^−8^^a^	0.013^a^	M ​> ​F
	−2, 2, −12	4.91		0.042	
L Posterior insula/planum temporale	−45, −11, −9	5.15	2.3 ​× ​10^−7^^a^	0.014^a^	M ​> ​F
	−32, −23, 14	4.95		0.035	
	−44, −14, 8	4.93		0.038	
L & R Precuneus/Posterior cingulate	−5, −38, 5	5.13	4.8 ​× ​10^−8^^a^	0.015^a^	F ​> ​M
	−2, −41, 30	5.04		0.023	
	2, −48, 27	5.00		0.028	
L & R frontal poles	−8, 62, 8	5.08	7.0 ​× ​10^−4^^a^	0.020	
R Fusiform	36, −24, −26	4.79	0.0030^a^		M ​> ​F
R Middle frontal	39, 57, 3	4.67	2.0 ​× ​10^−4^^a^		M ​> ​F

L: Left R: Right M: Male F: Female.

a Significant after Bonferroni correction.

### 3.1 Sex differences

We first delineated sex differences in brain structure within our cohort. Males had increased GM volume compared to females in regions situated in the temporal lobes, insula, hypothalamus and cerebellum. Females showed relatively greater GM volume than males around the superior parietal lobe and superior temporal and ventrolateral prefrontal cortices. GM regions with significant differences between males and females are presented in Table 1. WM regions are presented in Table 2.

### 3.2 Regions associated with AR(CAG)n

We next sought to determine regions in which GM volume is correlated with AR(CAG)n. Modulated GM maps were smoothed and compared by VBM. Initial analysis using a median split (>17 repeats) showed that males with long repeats (L) had greater GM density in the temporal lobes bilaterally; females who were long repeat homozygotes (LL) and heterozygotes (SL) had greater posterior insula GM density bilaterally than short repeat homozygotes (SS); and LL females had greater posterior insula GM density bilaterally than SL and SS females. The only sex by genotype interaction was in the right posterior insula, where LL females showed significantly greater GM density, while males showed genotypic differences.

GM density in the temporal lobes, orbitofrontal and sub-genual anterior cingulate cortices bilaterally and the right insula (p ​< ​0.01, FWE-corrected) was significantly correlated with AR(CAG)n. Within the temporal lobe there were a number of distinct clusters that were positively correlated with number of repeats. Also, positively correlated were the right supra-genual cingulate, right inferior cerebellum, left dorsomedial prefrontal cortex and right inferior parietal (p ​< ​0.05, FWE-corrected) as well as the left inferior cerebellum (p ​< ​0.001, cluster-level corrected). The GM regions with positive correlation with AR(CAG)n are presented in Table 1. The right superior parietal lobule was negatively correlated in GM volume with number of repeats (p ​< ​0.01, FWE-corrected).

The bilateral anterior corona radiata, left superior fasciculus and thalamus were positively correlated with AR(CAG)n (p ​< ​0.05, FWE-corrected). The WM regions with positive correlation with AR(CAG)n are presented in Table 2.

### 3.3 Regions associated with ESR1(TA)n

Initial analysis using a median split grouping ESR1(TA)n individuals with short repeat count (S) and long repeat count (L) alleles showed that in males, SS individuals had greater GM density than SL and LL, and SS and SL had greater GM density than LL in ventral prefrontal cortex, anterior cingulate cortex and temporal lobes. GM density correlated negatively with the number of TA repeats in these regions. GM regions with negative correlation with ESR1(TA)n are presented in Table 3 (p ​< ​0.05, FWE-corrected). The Left superior longitudinal fasciculus (p ​< ​0.001) and Left inferior longitudinal fasciculus (p ​< ​0.05) were significantly negatively correlated with ESR1(TA)n (Table 4, p ​< ​0.05, FWE-corrected).

### 3.4 Regions associated with ESR2(CA)n

Initial analysis using a median split (31=< S allele, >31 repeats L allele) showed no differences between in GM density in males, and in females greater GM density in posterior insula, temporal lobes and rostral prefrontal (p ​< ​0.01) in SS compared to SL/LL and in posterior insula, temporal lobes, rostral prefrontal, posterior cingulate and hypothalamus in SS/SL compared to LL.

Similar regions were associated with ESR2(CA)n as with AR(CAG)n in particular in the temporal lobes involving the parahippocampal and superior temporal gyri, hypothalamus, orbitofrontal cortex, insula, anterior cingulate and lateral prefrontal cortex. This was found to be primarily driven by an association in women, but not in men, showing a gender interaction with ESR2(CA)n. GM volume was negatively associated with ESR2(CA)n. The GM regions with negative correlation with ESR2(CA)n are presented in Table 5 (p ​< ​0.05, FWE-corrected). No WM regions significantly associated with ESR2(CA)n were found.

### 3.5 Replication cohort

We sought to determine whether the same regional associations were present for each polymorphism in an independent replication cohort. We replicated many of our findings in the discovery sample despite the small sample size of the replication sample. In the replication sample, AR(CAG)n length was positively correlated with GM volume in the temporal lobes bilaterally including the medial temporal lobe after correction for family-wise error (FWE) on the cluster level. The orbitofrontal cortex and hypothalamus were also positively correlated with GM volume, although the correlation did not survive family-wise error correction for multiple testing. Bilateral corticopontine tract volume was significantly positively associated with AR(CAG)n length in the replication cohort.

We replicated the negative association of ESR1(TA)n length with bilateral cerebellar and hypothalamic GM volume and bilateral superior longitudinal fasciculus (SLF) and inferior longitudinal fasciculus (ILF) WM volume and replicated the **negative association of ESR2(CA)n length with GM volume in the insula bilaterally as well as frontal and temporal cortex.**The full tables of significant findings in the replication cohort can be found in Supplementary Information.

## 4 Discussion

We found significant, replicable brain volume differences associated with three microsatellite polymorphisms of the sex hormone receptors that appeared mostly in regions of brain sex differences identified in prior research. This is of note because these hormone pathways are central to the development of brain sex differences and point to a possible mechanism for these differences through their opposing influences.

AR(CAG)n positively correlated with GM and WM in regions of brain sex differences. In adolescent males, it has been shown that in those with lower AR(CAG)n, individuals with high testosterone have greater WM volume than those with low testosterone (Perrin et al., 2008). However, in our sample, the effect of AR(CAG)n was present in both males and females. Another study showed that lower AR(CAG)n in men with low testosterone is associated with Alzheimer’s disease (Lehmann et al., 2003), suggesting that high expression of androgen receptors in the absence of androgen has detrimental effects. In Kennedy’s disease, where the mutation in AR(CAG)n has excessively long repeats increased androgens are detrimental (Kinirons et al., 2008). Taken together, the evidence suggests that androgen receptor occupancy may relate to GM volume. This relationship is likely to be mediated by a number of mechanisms, from a specific detrimental effect of unoccupied androgen receptors to the relative influence of the androgen and estrogen receptors in those regions.

Most of the areas of greatest association with AR(CAG)n were also different between the sexes. WM regions associated with AR(CAG)n were regions we found to be larger in females. The anterior cingulate cortex and the medial prefrontal cortex was also larger in females. On the other hand, the temporal and lateral prefrontal cortex was larger in males. However, while these GM regions consistently demonstrated a positive correlation with longer AR(CAG)n, the regions were not always larger in one sex than another. This simply suggests that there are multiple possibly opposing influences mediating brain sex differences that may have different regional effects.

ESR1(TA)n negatively correlated with GM in the temporal cortex, anterior cingulate, hypothalamus and prefrontal regions and WM in the corona radiata, cingulum and ILF in the occipital cortex. Longer TA repeats have been associated with increased anxiety in men (Comings et al., 1999) and postpartum depression in women (Pinsonneault et al., 2013); as well as increased psychoticism and irritability in women (Westberg et al., 2003) and decreased harm avoidance in both sexes (Gade-Andavolu et al., 2009). This would be in keeping with smaller regional volume in these regions with longer TA repeats. ESR1 genotype has been associated with WM lesions in elderly women (Ma et al., 2009) and GM volume in the cerebellum, temporal cortex, middle frontal gyrus and occipital lobe (Boccardi et al., 2008) and the planum temporale (Guadalupe et al., 2015) similar to what was found here with ESR1(TA)n. However we also found an association with the hypothalamus and anterior cingulate, regions also associated with AR(CAG)n.

ESR2(CA)n was negatively associated with GM volume in the posterior insula, hypothalamus, prefrontal regions, posterior cingulate and fusiform only in females and not in males. In women, shorter ESR2(CA)n repeats are associated with increased bone mineral density suggesting higher expression of estrogen beta receptors (Ichikawa et al., 2005). This would be consistent with the negative association and the specificity of the association with women. There is also some evidence implicating presence of lower ESR2(CA)n in depression in postmenopausal women (Takeo et al., 2005) and adolescent girls (Geng et al., 2007).

Overall, of all the regions of the brain containing sex differences, there appear to be a subset of common regions sensitive to the influence of sex hormone receptor polymorphism including the hypothalamus, temporal cortex, prefrontal cortex, anterior cingulate cortex and posterior insula.

The hypothalamus has long been thought to be the seat of brain sex differences. Conspicuous differences in size have been found in the preoptic hypothalamic area between sexes and in homosexual vs heterosexual individuals (Swaab et al., 1995). Androgen receptor staining has been shown to be more intense in men than women in the medial mammillary and lateral mammillary nuclei of the hypothalamus (Fernandez-Guasti et al., 2000). Kisspeptin, a peptide shown to be crucial for puberty, is concentrated within the anteroventral periventricular nucleus and the preoptic periventricular nucleus of the hypothalamus (Clarkson et al., 2006). Functionally, it has been shown sexual dimorphism in hypothalamic circuits where tyrosine-hydroxylase expressing neurons in the anteroventral periventricular nucleus control maternal care and oxytocin secretion in females and suppress intermale aggression in males (Scott et al., 2015). There are also significant differences in hypothalamic activation between homosexual men and heterosexual men (Savic et al., 2005).

The temporal lobes appear to be a key area associated with brain sex differences and a number of studies provide strong support for prominent sex hormone influences on this part of the brain (Janowsky, 2006). Some reports suggest faster age-related temporal lobe atrophy in men (Cowell et al., 1994), with higher local cortical glucose metabolism in men than women (Gur et al., 1995). Given the importance of the temporal lobes in the development of Alzheimer’s and the importance of sex hormones and sex hormone receptor polymorphisms in risk for Alzheimer’s (Carter et al., 2012; Lehmann et al., 2003), the association would be relevant for future studies elucidating this link. The temporal lobe has been previously implicated in sex differences in spatial abilities (Maguire et al., 1999) and visuo-spatial working memory during mental rotation (Schoning et al., 2007) and prenatal testosterone has been shown to improve spatial learning and memory in the temporal and frontal lobes in rats (Gurzu et al., 2008).

Medial prefrontal lesions have been shown to modify sexual and maternal behaviours in rats (Afonso et al., 2007), while estrogen has been shown to mediate sex differences in stress responses within the prefrontal cortex (Shansky et al., 2004). Stress and emotion related activity in the prefrontal cortex and anterior cingulate cortex have been shown to be modulated by the menstrual cycle (Goldstein et al., 2010) and to be impaired in premenstrual dysphoria (Comasco et al., 2014) suggesting that sex hormone influences in these regions may have implications for brain sex differences in mood disorders.

Of particular note, the posterior insula was a region found to be associated with sex hormone treatment in transgender individuals (Spizzirri et al., 2018). The insula together with the anterior cingulate cortex are core components of the salience network and it modulates autonomic reactivity in response to salient stimuli (Menon et al., 2010). Testosterone has been shown to interact with MAOA genotype in reducing harm avoidance during financial decision-making by blunting insula activation (Wagels et al., 2017).

There are some limitations of our findings that we present below. We could not have controlled for every confounding variable, and outside the major ones we controlled for in our general linear model, sex, age, gender, and scanner type, we did not account for serum oestrogen and androgen levels, menstrual cycles, years of education, handedness, and sexual orientation. We expect investigation into these variables to reveal additional interactions with genotype and phenotype in future studies. Additionally, we only studied microsatellite polymorphisms in this study without performing genotyping for relevant single nucleotide polymorphisms in the candidate genes, which could have further validated the findings in the study. Given that the study was performed in a healthy population, any implications for disease would require further validation with a relevant patient population. Another limitation to the study was that the images in the replication sample were acquired in a different scanner and had a relatively small sample size compared to the discovery dataset, which may have had an impact on the study. Nonetheless, the findings of the study represent a significant contribution to the understanding of sex differences in the brain and the contribution of sex hormone receptor expression. Furthermore, we demonstrate a dose effect of microsatellite length which is a novel representation of an understudied area of genomics.

## CRediT authorship contribution statement

**Geoffrey Chern-Yee Tan:** Conceptualization, Data curation, Formal analysis, Project administration, Validation, Writing - original draft, Writing - review & editing. **Carlton Chu:** Formal analysis. **Yu Teng Lee:** Writing - review & editing. **Clarence Chih King Tan:** Writing - review & editing. **John Ashburner:** Supervision, Funding acquisition, Writing - review & editing. **Nicholas W. Wood:** Supervision, Resources, Writing - review & editing. **Richard SJ. Frackowiak:** Supervision, Funding acquisition, Resources, Writing - review & editing.

## References

[bib1] Afonso V.M., Sison M., Lovic V., Fleming A.S. (2007). Medial prefrontal cortex lesions in the female rat affect sexual and maternal behavior and their sequential organization. Behav. Neurosci..

[bib3] Ashburner J. (2007). A fast diffeomorphic image registration algorithm. Neuroimage.

[bib115] Ashburner J., Friston K.J. (2005). Unified segmentation. Neuroimage.

[bib4] Becherini L., Gennari L., Masi L., Mansani R., Massart F., Morelli A. (2000). Evidence of a linkage disequilibrium between polymorphisms in the human estrogen receptor α gene and their relationship to bone mass variation in postmenopausal Italian women. Hum. Mol. Genet..

[bib5] Beilin J., Ball E., Favaloro J., Zajac J. (2000). Effect of the androgen receptor CAG repeat polymorphism on transcriptional activity: specificity in prostate and non-prostate cell lines. J. Mol. Endocrinol..

[bib7] Boccardi M., Scassellati C., Ghidoni R., Testa C., Benussi L., Bonetti M. (2008). Effect of the XbaI polymorphism of estrogen receptor alpha on postmenopausal gray matter. Neurosci. Lett..

[bib9] Butovskaya M.L., Lazebny O.E., Vasilyev V.A., Dronova D.A., Karelin D.V., Audax Z P Mabulla (2015). Androgen receptor gene polymorphism, aggression, and reproduction in tanzanian foragers and pastoralists. PLoS One.

[bib10] Carter C.L., Resnick E.M., Mallampalli M., Kalbarczyk A. (2012). Sex and gender differences in Alzheimer’s disease: recommendations for future research. J. Wom. Health.

[bib11] Celec P., Tretinárová D., Minárik G., Ficek A., Szemes T., Lakatošová S. (2013). Genetic polymorphisms related to testosterone metabolism in intellectually gifted boys. PLoS One.

[bib12] Chamberlain N.L., Driver E.D., Miesfeld R.L. (1994). The length and location of CAG trinucleotide repeats in the androgen receptor N-terminal domain affect transactivation function. Nucleic Acids Res..

[bib14] Choong C.S., Kemppainen J.A., Zhou Z.X., Wilson E.M. (1996). Reduced androgen receptor gene expression with first exon CAG repeat expansion. Mol. Endocrinol..

[bib15] Clarkson J., Herbison A.E. (2006). Postnatal development of kisspeptin neurons in mouse hypothalamus; brain sex differences and projections to gonadotropin-releasing hormone neurons. Endocrinology.

[bib16] Comasco E., Hahn A., Ganger S., Gingnell M., Bannbers E., Oreland L. (2014). Emotional fronto-cingulate cortex activation and brain derived neurotrophic factor polymorphism in premenstrual dysphoric disorder. Hum. Brain Mapp..

[bib17] Comings D.E., Muhleman D., Johnson P., MacMurray J.P. (1999). Potential role of the estrogen receptor gene (ESR1) in anxiety. Mol. Psychiatr..

[bib18] Cosgrove K.P., Mazure C.M., Staley J.K. (2007). Evolving knowledge of sex differences in brain structure, function, and chemistry. Biol. Psychiatr..

[bib19] Cowell P.E., Turetsky B.I., Gur R.C., Grossman R.I., Shtasel D., Gur R.E. (1994). Sex differences in aging of the human frontal and temporal lobes. J. Neurosci..

[bib20] Darnai G., Plózer E., Perlaki G., Orsi G., Nagy S.A., Horváth R. (2016). 2D:4D finger ratio positively correlates with total cerebral cortex in males. Neurosci. Lett..

[bib22] Deichmann R. (2006). Fast structural brain imaging using an MDEFT sequence with a FLASH–EPI hybrid readout. Neuroimage.

[bib23] Deichmann R., Schwarzbauer C., Turner R. (2004). Optimisation of the 3D MDEFT sequence for anatomical brain imaging: technical implications at 1.5 and 3 T. Neuroimage.

[bib24] Deng Y., Wang J., Wang L., Du Y. (2017). Androgen receptor gene CAG repeat polymorphism and ovarian cancer risk: a meta-analysis. BioSci. Trends.

[bib25] Eastwood H., Brown K.M.O., Markovic D., Pieri L.F. (2002). Variation in the ESR1 and ESR2 genes and genetic susceptibility to anorexia nervosa. Mol. Psychiatr..

[bib26] Fernández-Guasti A., Kruijver F.P., Fodor M., Swaab D.F. (2000). Sex differences in the distribution of androgen receptors in the human hypothalamus. J. Comp. Neurol..

[bib27] Gade-Andavolu R., MacMurray J., Comings D.E., Calati R., Chiesa A., Serretti A. (2009). Association between the estrogen receptor TA polymorphism and harm avoidance. Neurosci. Lett..

[bib28] Garcia M., Rochefort H. (1979). Evidence and characterization of the binding of two 3H-labeled androgens to the estrogen receptor. Endocrinology.

[bib114] Gardiner S.L., Harder A.V.E., Campman Y.J.M., Trompet S., Gussekloo J., van Belzen M.J. (2019). Repeat length variations in ATXN1 and AR modify disease expression in Alzheimer’s disease. Neurobiology of Aging.

[bib29] Geng Y., Su Q., Su L., Chen Q., Ren G. (2007). Comparison of the polymorphisms of androgen receptor gene and estrogen α and β gene between adolescent females with first-onset major depressive disorder and controls. Int. J. Neurosci..

[bib30] Giovannucci E., Platz E.A., Stampfer M.J., Chan A., Krithivas K., Kawachi I., Kantoff P.W. (1999). The CAG repeat within the androgen receptor gene and benign prostatic hyperplasia. Urology.

[bib31] Goldstein J.M., Jerram M., Abbs B., Whitfield-Gabrieli S., Makris N. (2010). Sex differences in stress response circuitry activation dependent on female hormonal cycle. J. Neurosci..

[bib32] Good C.D., Johnsrude I., Ashburner J., Henson R.N.A., Friston K.J., Frackowiak R.S.J. (2001). Cerebral asymmetry and the effects of sex and handedness on brain structure: a voxel-based morphometric analysis of 465 normal adult human brains. Neuroimage.

[bib33] Gorka A.X., Norman R.E., Radtke S.R., Carré J.M., Hariri A.R. (2015). Anterior cingulate cortex gray matter volume mediates an association between 2D:4D ratio and trait aggression in women but not men. Psychoneuroendocrinology.

[bib34] Gräns H., Nilsson M., Dahlman-Wright K., Evengard B. (2007). Reduced levels of oestrogen receptor β mRNA in Swedish patients with chronic fatigue syndrome. J. Clin. Pathol..

[bib35] Guadalupe T., Zwiers M.P., Wittfeld K., Teumer A., Vasquez A.A., Hoogman M. (2015). Asymmetry within and around the human planum temporale is sexually dimorphic and influenced by genes involved in steroid hormone receptor activity. Cortex.

[bib36] Gur R.C., Turetsky B.I., Matsui M., Yan M., Bilker W., Hughett P., Gur R.E. (1999). Sex differences in brain gray and white matter in healthy young adults: correlations with cognitive performance. J. Neurosci..

[bib37] Gurzu C., Artenie V., Hritcu L., Ciobica A. (2008). Prenatal testosterone improves the spatial learning and memory by protein synthesis in different lobes of the brain in the male and female rat. Cent. Eur. J. Biol..

[bib38] Gustafson D.R., Wen M.J., Koppanati B.M. (2003). Androgen receptor gene repeats and indices of obesity in older adults. Int. J. Obes..

[bib39] Hahn A., Kranz G.S., Sladky R., Kaufmann U., Ganger S., Hummer A. (2016). Testosterone affects language areas of the adult human brain: testosterone affects language areas. Hum. Brain Mapp..

[bib41] Hao Y., Montiel R., Li B., Huang E., Zeng L., Huang Y. (2010). Association between androgen receptor gene CAG repeat polymorphism and breast cancer risk: a meta-analysis. Breast Canc. Res. Treat..

[bib42] Hedges V.L., Ebner T.J., Meisel R.L., Mermelstein P.G. (2012). The cerebellum as a target for estrogen action. Front. Neuroendocrinol..

[bib44] Hines M. (2010). Sex-related variation in human behavior and the brain. Trends Cognit. Sci..

[bib45] Hines M. (2011). Gender development and the human brain. Annu. Rev. Neurosci..

[bib46] Ichikawa S., Koller D.L., Peacock M., Johnson M.L., Lai D., Hui S.L. (2005). Polymorphisms in the estrogen receptor β (ESR2) gene are associated with bone mineral density in Caucasian men and women. J. Clin. Endocrinol. Metabol..

[bib47] Janowsky J.S. (2006). The role of androgens in cognition and brain aging in men. Neuroscience.

[bib48] Kantarci K., Tosakulwong N., Lesnick T.G., Zuk S.M., Gunter J.L., Gleason C.E. (2016). Effects of hormone therapy on brain structure: a randomized controlled trial. Neurology.

[bib49] Kinirons P., Rouleau G.A. (2008). Administration of testosterone results in reversible deterioration in Kennedy’s disease. J. Neurol. Neurosurg. Psychiatr..

[bib50] Kumar R., Atamna H., Zakharov M.N., Bhasin S., Khan S.H., Jasuja R. (2011). Role of the androgen receptor CAG repeat polymorphism in prostate cancer, and spinal and bulbar muscular atrophy. Life Sci..

[bib51] La Spada A.R.L., Wilson E.M., Lubahn D.B., Harding A.E., Fischbeck K.H. (1991). Androgen receptor gene mutations in X-linked spinal and bulbar muscular atrophy. Nature.

[bib52] La Spada A.R., Roling D.B., Harding A.E., Warner C.L., Speigel R., Hausmanowa- Petrusewicz I. (1992). Meiotic stability and genotype - phenotype correlation of the trinucleotide repeat in X-linked spinal and bulbar muscular atrophy. Nat. Genet..

[bib53] Laflamme N., Nappi R.E., Drolet G., Labrie C., Rivest S. (1998). Expression and neuropeptidergic characterization of estrogen receptors (ERα and ERβ) throughout the rat brain: anatomical evidence of distinct roles of each subtype. J. Neurobiol..

[bib54] Langdahl B.L., Løkke E., Carstens M., Stenkjær L.L., Eriksen E.F. (2000). A TA repeat polymorphism in the estrogen receptor gene is associated with osteoporotic fractures but polymorphisms in the first exon and intron are not. J. Bone Miner. Res..

[bib55] Lehmann D.J., Butler H.T., Warden D.R., Combrinck M., King E., Nicoll J.A.R., Budge M.M., de Jager C.A., Hogervorst E., Esiri M.M., Ragoussis J. (2003). Association of the androgen receptor CAG repeat polymorphism with Alzheimer’s disease in men. Neurosci. Lett..

[bib56] Lenz K.M., McCarthy M.M. (2010). Organized for sex - steroid hormones and the developing hypothalamus. Eur. J. Neurosci..

[bib113] Lephart E.D. (1996). A review of brain aromatase cytochrome P450. Brain Research Reviews.

[bib57] Lombardo M.V., Ashwin E., Auyeung B., Chakrabarti B., Taylor K., Hackett G. (2012). Fetal testosterone influences sexually dimorphic gray matter in the human brain. J. Neurosci..

[bib58] Luders E., Narr K.L., Thompson P.M., Rex D.E., Woods R.P., DeLuca H., Toga A.W. (2006). Gender effects on cortical thickness and the influence of scaling. Hum. Brain Mapp..

[bib59] Ma S.L., Tang N.L., Tam C.W., Lui V.W., Lau E.S., Zhang Y.P. (2009). Polymorphisms of the estrogen receptor α (ESR1) gene and the risk of Alzheimer’s disease in a southern Chinese community. Int. Psychogeriatr..

[bib60] Maguire E.A., Burgess N., O’Keefe J. (1999). Human spatial navigation: cognitive maps, sexual dimorphism, and neural substrates. Curr. Opin. Neurobiol..

[bib61] Menon V., Uddin L.Q. (2010). Saliency, switching, attention and control: a network model of insula function. Brain Struct. Funct..

[bib62] Neufang S., Specht K., Hausmann M., Güntürkün O., Herpertz-Dahlmann B., Fink G.R., Konrad K. (2009). Sex differences and the impact of steroid hormones on the developing human brain. Cerebr. Cortex.

[bib63] Nguyen T. (2018). Developmental effects of androgens in the human brain. J*. Neuroendocrinol.*.

[bib64] Nguyen T., McCracken J., Ducharme S., Botteron K.N., Mahabir M., Johnson W. (2013). For the brain development cooperative group. Testosterone-related Cortical Maturation Across Childhood Adolesc. Cerebr. Cortex.

[bib65] Nilsson M., Naessén S., Dahlman I., Lindén Hirschberg A., Gustafsson J., Dahlman-Wright K. (2004). Association of estrogen receptor beta gene polymorphisms with bulimic disease in women. Mol. Psychiatr..

[bib66] Nowak N.T., Diamond M.P., Land S.J., Moffat S.D. (2013). Contributions of sex, testosterone, and androgen receptor CAG repeat number to virtual morris water maze performance. Psychoneuroendocrinology.

[bib67] Ogawa S., Emi M., Shiraki M., Hosoi T., Ouchi Y., Inoue S. (2000). Association of estrogen receptor β (ESR2) gene polymorphism with blood pressure. J. Hum. Genet..

[bib68] Paus T., Nawaz-Khan I., Leonard G., Perron M., Pike G.B., Pitiot A. (2010). Brain sex differences in the adolescent brain: role of testosterone and androgen receptor in global and local volumes of grey and white matter. Horm. Behav..

[bib69] Perrin J.S., Hervé P.Y., Leonard G., Perron M., Pike G.B., Pitiot A. (2008). Growth of white matter in the adolescent brain: role of testosterone and androgen receptor. J. Neurosci..

[bib70] Peters A.A., Buchanan G., Ricciardelli C., Bianco-Miotto T., Centenera M.M., Harris J.M. (2009). Androgen receptor inhibits estrogen receptor-α activity and is prognostic in breast cancer. Canc. Res..

[bib71] Pinsonneault J.K., Sullivan D., Sadee W., Soares C.N., Hampson E., Steiner M. (2013). Association study of the estrogen receptor gene ESR1 with postpartum depression—a pilot study. Arch. Wom. Ment. Health.

[bib72] Pirskanen M., Hiltunen M., Mannermaa A., Helisalmi S., Lehtovirta M., Hänninen T., Soininen H. (2005). Estrogen receptor beta gene variants ar associated with increased risk of Alzheimer’s disease in women. Eur. J. Hum. Genet..

[bib73] Prichard Z., Jorm A.F., Prior M., Sanson A., Smart D., Zhang Y. (2002). Association of polymorphisms of the estrogen receptor gene with anxiety-related traits in children and adolescents: a longitudinal study. Am. J. Med. Genet..

[bib74] Qin Z., Li X., Han P., Zheng Y., Liu H., Tang J. (2017). Association between polymorphic CAG repeat lengths in the androgen receptor gene and susceptibility to prostate cancer: a systematic review and meta-analysis. Medicine.

[bib75] Raznahan A., Lee Y., Stidd R., Long R., Greenstein D., Clasen L. (2010). Longitudinally mapping the influence of sex and androgen signaling on the dynamics of human cortical maturation in adolescence. Proc. Natl. Acad. Sci. Unit. States Am..

[bib76] Rexrode K.M., Ridker P.M., Hegener H.H., Buring J.E., Manson J.E., Zee R.Y.L. (2007). Polymorphisms and haplotypes of the estrogen receptor-β gene (ESR2) and cardiovascular disease in men and women. Clin. Chem..

[bib77] Ritchie S.J., Cox S.R., Shen X., Lombardo M.V., Reus L.M., Alloza C. (2018). Sex differences in the adult human brain: evidence from 5216 UK Biobank participants. Cerebr. Cortex.

[bib78] Roiser J.P., de Martino B., Tan G.C., Kumaran D., Seymour B., Wood N.W., Dolan R.J. (2009). A genetically mediated bias in decision making driven by failure of amygdala control. J. Neurosci..

[bib79] Ruigrok A.N.V., Salimi-Khorshidi G., Lai M., Baron-Cohen S., Lombardo M.V., Tait R.J., Suckling J. (2014). A meta-analysis of sex differences in human brain structure. Neurosci. Biobehav. Rev..

[bib80] Savic I., Berglund H., Lindström P. (2005). Brain response to putative pheromones in homosexual men. Proc. Natl. Acad. Sci. Unit. States Am..

[bib81] Scariano J.K., Simplicio S.G., Montoya G.D., Garry P.J., Baumgartner R.N. (2004). Estrogen receptor β dinucleotide (CA) repeat polymorphism is significantly associated with bone mineral density in postmenopausal women. Calcif. Tissue Int..

[bib82] Schöning S., Engelien A., Kugel H., Schäfer S., Schiffbauer H., Zwitserlood P. (2007). Functional anatomy of visuo-spatial working memory during mental rotation is influenced by sex, menstrual cycle, and sex steroid hormones. Neuropsychologia.

[bib83] Schuit S.C.E., van Meurs, Joyce B.J., Bergink A.P., van der Klift M., Fang Y., Leusink G. (2004). Height in pre- and postmenopausal women is influenced by estrogen receptor α gene polymorphisms. J. Clin. Endocrinol. Metabol..

[bib84] Scott N., Prigge M., Yizhar O., Kimchi T. (2015). A sexually dimorphic hypothalamic circuit controls maternal care and oxytocin secretion. Nature.

[bib85] Sebastian C.L., Roiser J.P., Tan G.C., Viding E., Wood N.W., Blakemore S.J. (2010). Effects of age and MAOA genotype on the neural processing of social rejection. Gene Brain Behav..

[bib86] Seiger R., Hahn A., Hummer A., Kranz G.S., Ganger S., Woletz M. (2016). Subcortical gray matter changes in transgender subjects after long-term cross-sex hormone administration. Psychoneuroendocrinology.

[bib87] Shansky R.M., Glavis-Bloom C., Lerman D., McRae P., Benson C., Miller K., Cosand L., Horvath T.L., Arnsten A.F.T. (2004). Estrogen mediates sex differences in stress-induced prefrontal cortex dysfunction. Mol. Psychiatr..

[bib88] Spizzirri G., Duran F.L.S., Chaim-Avancini T.M., Serpa M.H., Cavallet M., Pereira C.M.A. (2018). Grey and white matter volumes either in treatment-naïve or hormone-treated transgender women: a voxel-based morphometry study. Sci. Rep..

[bib89] Stonnington C.M., Tan G., Klöppel S., Chu C., Draganski B., Jack C.R. (2008). Interpreting scan data acquired from multiple scanners: a study with Alzheimer’s disease. Neuroimage.

[bib91] Swaab Dichk F., Hofman Michel A. (1995). Sexual differentiation of the human hypothalamus in relation to gender and sexual orientation. Trends Neurosci..

[bib92] Takeo C., Negishi E., Nakajima A., Ueno K., Tatsuno I., Saito Y. (2005). Association of cytosine-adenine repeat polymorphism of the estrogen receptor-β gene with menopausal symptoms. Gend. Med..

[bib93] Tan G.C., Doke T.F., Ashburner J., Wood N.W., Frackowiak R.S. (2010). Normal variation in fronto-occipital circuitry and cerebellar structure with an autism-associated polymorphism of CNTNAP2. Neuroimage.

[bib94] Tang L., Li J., Bao M., Xiang J., Chen Y., Wang Y. (2018). Genetic association between HER2 and ESR2 polymorphisms and ovarian cancer: a meta-analysis. OncoTargets Ther..

[bib95] Tsukamoto K., Inoue S., Hosoi T., Orimo H., Emi M. (1998). Isolation and radiation hybrid mapping of dinucleotide repeat polymorphism at the human estrogen receptor β locus. J. Hum. Genet..

[bib96] Vaillancourt K.L., Dinsdale N.L., Hurd P.L. (2012). Estrogen receptor 1 promoter polymorphism and digit ratio in men. Am. J. Hum. Biol..

[bib97] Valla J.M., Ceci S.J. (2011). Can sex differences in science be tied to the long reach of prenatal hormones? Brain organization theory, digit ratio (2D/4D), and sex differences in preferences and cognition. Perspect. Psychol. Sci..

[bib99] Wagels L., Votinov M., Radke S., Clemens B., Montag C., Jung S., Habel U. (2017). Blunted insula activation reflects increased risk and reward seeking as an interaction of testosterone administration and the MAOA polymorphism. Hum. Brain Mapp..

[bib100] Westberg L., Baghaei F., Rosmond R., Hellstrand M., Landén M., Jansson M. (2001). Polymorphisms of the androgen receptor gene and the estrogen receptor beta gene are associated with androgen levels in women. J. Clin. Endocrinol. Metabol..

[bib101] Westberg L., Melke J., Landén M., Nilsson S., Baghaei F., Rosmond R. (2003). Association between a dinucleotide repeat polymorphism of the estrogen receptor alpha gene and personality traits in women. Mol. Psychiatr..

[bib102] Westberg L., Håkansson A., Melke J., Niazi Shahabi H., Nilsson S., Buervenich S. (2004). Association between the estrogen receptor beta gene and age of onset of Parkinson’s disease. Psychoneuroendocrinology.

[bib103] Westberg L., Henningsson S., Landén M., Annerbrink K. (2009). Influence of androgen receptor repeat polymorphisms on personality traits in men. J. Psychiatr. Neurosci..

[bib104] Wierenga L.M., Bos M.G.N., Schreuders E., vd Kamp F., Peper J.S., Tamnes C.K., Crone E.A. (2018). Unraveling age, puberty and testosterone effects on subcortical brain development across adolescence. Psychoneuroendocrinology.

[bib105] Witte A.V., Savli M., Holik A., Kasper S., Lanzenberger R. (2010). Regional sex differences in grey matter volume are associated with sex hormones in the young adult human brain. Neuroimage.

[bib106] Wittmann B.C., Tan G.C., Lisman J.E., Dolan R.J., Düzel E. (2013). DAT genotype modulates striatal processing and long-term memory for items associated with reward and punishment. Neuropsychologia.

[bib107] Yaffe K., Edwards E.R., Lui L., Zmuda J.M., Ferrell R.E., Cauley J.A. (2003). Androgen receptor CAG repeat polymorphism is associated with cognitive function in older men. Biol. Psychiatr..

[bib108] Yamada Y., Ando F., Niino N., Shimokata H. (2005). Association of polymorphisms of the androgen receptor and klotho genes with bone mineral density in Japanese women. J. Mol. Med..

[bib109] Yu K., Rao N., Chen A., Fan L., Yang C., Shao Z. (2011). A systematic review of the relationship between polymorphic sites in the estrogen receptor-beta (ESR2) gene and breast cancer risk. Breast Canc. Res. Treat..

[bib110] Zhang K., Sejnowski T.J. (2000). A universal scaling law between gray matter and white matter of cerebral cortex. Proc. Natl. Acad. Sci. Unit. States Am..

[bib111] Zhao L., Gu C., Huang K., Fan W., Li L., Ye M. (2016). Association between oestrogen receptor alpha (ESR1) gene polymorphisms and endometriosis: a meta-analysis of 24 case-control studies. Reprod. Biomed. Online.

[bib112] Zubiaurre-Elorza L., Junque C., Gómez-Gil E., Guillamon A. (2014). Effects of cross-sex hormone treatment on cortical thickness in transsexual individuals. J. Sex. Med..

