# Supplementary material for: The influence of microsatellite polymorphisms in sex steroid receptor genes ESR1, ESR2 and AR on sex differences in brain structure
Source: Neuroimage. 2020 Nov 1;221:117087. doi: 10.1016/j.neuroimage.2020.117087 (PMC8960998; doi:10.1016/j.neuroimage.2020.117087)
Supplement: Multimedia component 1 [file mmc1.docx]

**Supplementary Information**

**List of Tables**

**Table S1:** Means and standard deviations for Caucasians within Discovery and Replication samples
**Table S2**: One-way ANOVA test for differences in mean between discovery and replication groups.
**Table S3**: GM Regions with differences between males and females.

**Table S4**: WM Regions with differences between males and females

**Table S5:** GM regions showing statistically significant positive correlations with AR(CAG)n in the replication sample.

**Table S6:** WM regions showing statistically significant positive correlations with AR(CAG)n in the replication sample. 
**Table S7:** GM regions showing statistically significant negative correlations with ESR1(TA)n in the replication sample.

**List of Figures
Figure 1.** Histogram of ESR1(TA) allele frequency, where the x-axis is the number of TA repeats **Figure 2.** Histogram of AR(CAG) allele frequency, where the x-axis is the number of CAG repeats **Figure 3.** Histogram of ESR2(CA) allele frequency, where the x-axis is the number of CA repeats

Presented are tables containing significant regions of grey and white matter regional differences with AR(CAG)n (Table 1 & Table 2), ESR1(TA)n (Table 3 & Table ), and grey matter differences in ESR2(TA)n (Table 5). No significant differences in white matter volume were found with ESR2(TA)n genotypes after family-wise error correction.

| Variable |  | N | Mean | Std. Deviation |
| --- | --- | --- | --- | --- |
| Male | Replication | 61 | 44% |  |
|  | Discovery | 173 | 49% |  |
|  | Total | 234 | 48% |  |
| Age in years | Replication | 61 | 30.5 | 11.7 |
|  | Discovery | 173 | 33.4 | 13.1 |
|  | Total | 234 | 32.6 | 12.8 |
| ESR1(TA)n averaged across alleles | Replication | 60 | 18.4 | 2.65 |
|  | Discovery | 170 | 18.8 | 2.71 |
|  | Total | 234 | 18.3 | 2.56 |
| ESR2(CA)n averaged across alleles | Replication | 61 | 30.5 | 1.55 |
|  | Discovery | 173 | 30.7 | 1.64 |
|  | Total | 234 | 30.5 | 1.63 |
| AR(CAG)n averaged across alleles | Replication | 61 | 18.3 | 2.54 |
|  | Discovery | 173 | 18.3 | 2.57 |
|  | Total | 234 | 18.3 | 2.55 |
| Grey volume in ml | Replication | 61 | 736.1 | 84.0 |
|  | Discovery | 173 | 743.8 | 72.2 |
|  | Total | 234 | 741.4 | 81.0 |
| White volume in ml | Replication | 61 | 464.4 | 49.6 |
|  | Discovery | 173 | 474.9 | 58.8 |
|  | Total | 234 | 472.2 | 56.6 |

**Table S1**: Means and standard deviations for Caucasians within Discovery and Replication samples

| **ANOVA** | | | | | | |
| --- | --- | --- | --- | --- | --- | --- |
|  |  | Sum of Squares | df | Mean Square | F | Sig. |
| Male | Between Groups | .107 | 1 | .107 | .426 | .515 |
|  | Within Groups | 58.286 | 232 | .251 |  |  |
|  | Total | 58.393 | 233 |  |  |  |
| Age | Between Groups | 381.173 | 1 | 381.173 | 2.336 | .128 |
|  | Within Groups | 37861.932 | 232 | 163.198 |  |  |
|  | Total | 38243.105 | 233 |  |  |  |
| ESR(TA) average length | Between Groups | 6.139 | 1 | 6.139 | .844 | .359 |
|  | Within Groups | 1659.161 | 228 | 7.277 |  |  |
|  | Total | 1665.300 | 229 |  |  |  |
| ESR2(CA) average length | Between Groups | 1.708 | 1 | 1.708 | .651 | .420 |
|  | Within Groups | 608.403 | 232 | 2.622 |  |  |
|  | Total | 610.111 | 233 |  |  |  |
| AR(CAG) average length | Between Groups | .020 | 1 | .020 | .003 | .956 |
|  | Within Groups | 1524.721 | 232 | 6.572 |  |  |
|  | Total | 1524.740 | 233 |  |  |  |
| Grey volume in ml | Between Groups | 1770.617 | 1 | 1770.617 | .269 | .605 |
|  | Within Groups | 1528117.083 | 232 | 6586.712 |  |  |
|  | Total | 1529887.700 | 233 |  |  |  |
| White volume in ml | Between Groups | 4956.842 | 1 | 4956.842 | 1.550 | .214 |
|  | Within Groups | 741803.863 | 232 | 3197.430 |  |  |
|  | Total | 746760.704 | 233 |  |  |  |

**Table S2**: One-way ANOVA test for differences in mean between discovery and replication groups.

**There were no significant differences between the discovery and replication samples across the variables used in the construction of the design matrix.*

| **GM Regions with differences between males and females** | | | | | |
| --- | --- | --- | --- | --- | --- |
| Region | [x, y, z]  MNI | Z-score | p-value, cluster, FWE-corrected | p-value, peak, FWE-corrected | Direction of sex difference |
| R Medial Temporal  R Insula | 35, -2, -26  38, 6, -17 | 6.63  6.52 | 1.6×10^-10^ | 1.0×10^-6^  2.0×10^-6^ | M>F |
| L Medial Temporal  L Insula  L Hypothalamus  R Hypothalamus | -35, -3 -26  -36, 5, -18  -5, -2, -5  8, -2, -5 | 6.60  5.93  6.09  5.82 | 1.4×10^-11^ | 1.2×10^-6^ | M>F |
| R Cerebellar hemisphere  Anterior Cerebellum | 57, -66, -18  24, -65, -44  6, -39, -18 | 5.75  5.67  5.46 | 2.0×10^-7^ | 1.7×10^-4^  2.6×10^-4^  8.0×10^-4^ | M>F |
| L Cerebellar hemisphere | -39, -85, -23  -23 -65 -44 | 6.08  5.88 | 9.5×10^-5^ | 2.8×10^-5^ | M>F |
| L Superior Parietal  R Anterior Cingulate  L Anterior Cingulate  R Superior Parietal | -14 -30 72  8, 17, 39  -6, 26, 33  15, -29, 74 | 4.94  4.21  4.06  3.94 | 2.8×10^-4^ | 0.018 | F>M |
| Occipital cortex | 0, -68, 27 | 4.91 | 6.4×10^-4^ | 0.011 | F>M |

L: Left R: Right M: Male F: Female

**Table S3**. GM Regions with differences between males and females.

| **WM Regions with differences between males and females** | | | | | |
| --- | --- | --- | --- | --- | --- |
| Region | [x, y, z]  MNI | Z-score | p-value, cluster, FWE-corrected | p-value, peak, FWE-corrected | Direction of sex difference |
| Infundibulum/Pituitary | -3 2 -21 | 5.32 |  | 0.17 | F>M |
| L Anterior corona radiata, L SLF  L Cingulum | -29, 5, 39  -14 -17 39 | 4.52  3.58 | 0.039 |  | F>M |
| L ILF | -29, -77, 12 | 4.04 | 0.038 |  | F>M |
| R Anterior corona radiata, R SLF, cingulum | 39, -24, 45 | 3.74 | 0.006 |  | F>M |
| R ILF | 42 -15 -9 | 3.51 | 0.044 |  | F>M |
| L insula/arcuate fasciculus | 32 26 -2 | 5.02 |  | 0.025 | M>F |
| L Anterior temporal | -47 12 -35 | 4.72 | 2.7×10^-6^ |  | M>F |
| L Precuneus | -6 -44 75 | 4.35 | 0.0023 |  | M>F |

L: Left R: Right M: Male F: Female ILF: Inferior Longitudinal Fasciculus SLF: Superior Longitudinal Fasciculus

**Table S4**. WM Regions with differences between males and females

| **GM Regions with positive correlation with AR(CAG)n** | | | | | |
| --- | --- | --- | --- | --- | --- |
| Region | [x, y, z]  MNI | Z-score | p-value, cluster , FWE-corrected | p-value, peak, FWE-corrected | Conjunction with sex differences |
| L Middle and inferior temporal gyri | -47, -20, -30 | 4.40 | 1.4×10^-4^ | 0.386 | M>F |
|  | -17, 3, -45 | 4.33 |  | 0.470 |  |
| Left Temporal pole | -45, 14, -36 | 4.10 |  | 0.771 | M>F |
| R Middle and inferior temporal gyri | 62, -17, -32 | 3.98 | 0.002 | 0.890 | M>F |
|  | 69, -15, -20 | 3.96 |  | 0.904 | M>F |
| R superior temporal gyrus | 66, -8, -9 | 3.86 |  | 0.964 | M>F |

L: Left R: Right M: Male F: Female

**Table S3.** GM regions showing statistically significant positive correlations with AR(CAG)n in the replication sample.

| **WM Regions with positive correlation with AR(CAG)n** | | | | | |
| --- | --- | --- | --- | --- | --- |
| Region | [x, y, z]  MNI | Z-score | p-value, cluster, FWE-corrected | p-value, peak, FWE-corrected | Conjunction with sex differences |
| L SLF | -29, -24, 23 | 3.92 | 0.002 | 0.956 | F>M |
| R Cingulum | 12, -24, 35 | 3.57 |  | 1 | F>M |
| L Corticopontine tract | -14, -32, 33 | 3.29 |  | 1 | M>F |
| Midbrain | 11, -21, -17 | 3.56 | 0.015 | 1 | M>F |
| Right Brainstem | -8, -11, -11 | 3.13 |  | 1 | M>F |
| Left Brainstem | -8, -18, -18 | 3.05 |  | 1 | M>F |

L: Left R: Right M: Male F: Female SLF: Superior Longitudinal Fasciculus

**Table S4.** WM regions showing statistically significant positive correlations with AR(CAG)n in the replication sample.

| **GM Regions with negative correlation with ESR1(TA)n** | | | | | |
| --- | --- | --- | --- | --- | --- |
| Region | [x, y, z]  MNI | Z-score | p-value, cluster, FWE-corrected | p-value, peak, FWE-corrected | Conjunction with sex differences |
| R Cerebellum  L Cerebellum | 29 -53 -27  -18 -65 -27 | 4.34  3.32 | 0.017 | 1 | M>F |
| L Hypothalamus | -3 0 -11 | 3.20 | 0.021 (after small volume correction) | 1 | M>F |

L: Left R: Right M: Male F: Female

**Table S5.** GM regions showing statistically significant negative correlations with ESR1(TA)n in the replication sample.

| **WM Regions with negative correlation with ESR1(TA)n** | | | | | |
| --- | --- | --- | --- | --- | --- |
| Region | [x, y, z]  MNI | Z-score | p-value, cluster, FWE-corrected | p-value, peak, FWE-corrected | Conjunction with sex differences |
| R SLF | 26, -18, 39 | 5.67 | 5.5×10^-7^ | 0.002 | F>M |
| R SLF | 30, -15, 32 | 5.58 |  | 0.003 | F>M |
| R ILF | 38, -5, 8 | 4.89 |  | 0.075 | F>M |
| L SLF | -36, -15, 24 | 5.54 | 3.0×10^-4^ | 0.004 | F>M |
| L ILF | -26, -14, 32 | 5.43 |  | 0.006 | F>M |
|  | -35, -21, -6 | 4.38 | 0.003 | 0.457 | F>M |
|  | -29, -74, -3 | 4.35 | 0.030 | 0.504 | F>M |

R: Right M: Male F: Female ILF: Inferior Longitudinal Fasciculus SLF: Superior Longitudinal Fasciculus

**Table S6.** WM regions showing statistically significant positive correlations with ESR1(TA)n in the replication sample.

| **GM Regions with negative correlation with ESR2(TA)n** | | | | | |
| --- | --- | --- | --- | --- | --- |
| Region | [x, y, z]  MNI | Z-score | p-value, cluster, FWE-corrected | p-value, peak, FWE-corrected | Conjunction with sex differences |
| R Frontal pole | 29, 50, -6 | 5.49 | 0.065 | 0.004 | M>F |
| R insula | 44, 21, -11 | 4.85 | 0.091 | 0.077 | M>F |
| L insula | -44, -18, 27 | 4.33 | 0.003 | 0.469 | M>F |
| L temporal | -63, -17, -11 | 4.33 | 0.119^#^ | 0.470 | M> F |

R: Right M: Male F: Female 
# Significant for FDR correction

**Table S7.** GM regions showing statistically significant negative correlations with ESR2(TA)n in the replication sample.


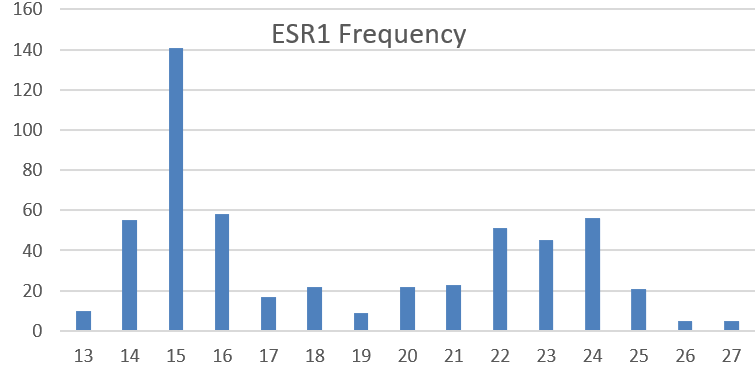


**Figure 1.** Histogram of ESR1(TA) allele frequency, where the x-axis is the number of TA repeats

**Figure 2.** Histogram of AR(CAG) allele frequency, where the x-axis is the number of CAG repeats

**Figure 3.** Histogram of ESR2(CA) allele frequency, where the x-axis is the number of CA repeats
